# Combining adult with pediatric patient data to develop a clinical decision support tool intended for children: leveraging machine learning to model heterogeneity

**DOI:** 10.1186/s12911-022-01827-4

**Published:** 2022-03-29

**Authors:** Paul Sabharwal, Jillian H. Hurst, Rohit Tejwani, Kevin T. Hobbs, Jonathan C. Routh, Benjamin A. Goldstein

**Affiliations:** 1 Department of Computer Science, Duke University, Durham, NC USA; 2 Children’s Health and Discovery Initiative, Department of Pediatrics, Duke University, Durham, NC USA; 3 Division of Infectious Diseases, Department of Pediatrics, Duke University, Durham, NC USA; 4 Division of Urology, Department of Surgery, Duke University, Durham, NC USA; 5 Department of Biostatistics and Bioinformatics, Duke University, 2424 Erwin Road, Durham, NC 27705 USA

**Keywords:** Predictive modeling, Electronic health records, Machine learning, Pediatrics

## Abstract

**Background:**

Clinical decision support (CDS) tools built using adult data do not typically perform well for children. We explored how best to leverage adult data to improve the performance of such tools. This study assesses whether it is better to build CDS tools for children using data from children alone or to use combined data from both adults and children.

**Methods:**

Retrospective cohort using data from 2017 to 2020. Participants include all individuals (adults and children) receiving an elective surgery at a large academic medical center that provides adult and pediatric services. We predicted need for mechanical ventilation or admission to the intensive care unit (ICU). Predictor variables included demographic, clinical, and service utilization factors known prior to surgery. We compared predictive models built using machine learning to regression-based methods that used a pediatric or combined adult-pediatric cohort. We compared model performance based on Area Under the Receiver Operator Characteristic.

**Results:**

While we found that adults and children have different risk factors, machine learning methods are able to appropriately model the underlying heterogeneity of each population and produce equally accurate predictive models whether using data only from pediatric patients or combined data from both children and adults. Results from regression-based methods were improved by the use of pediatric-specific data.

**Conclusions:**

CDS tools for children can successfully use combined data from adults and children if the model accounts for underlying heterogeneity, as in machine learning models.

## Background

Clinical decision support (CDS) tools are increasingly being used to assist providers during routine clinical care and for treatment decisions. While tools that are developed for specialized environments or specific populations often have better performance [1], there is a logistical cost to implementing and maintaining multiple models. The development of population-specific CDS tools requires significant additional effort for development, implementation, training, and maintenance. Moreover, development of subgroup-specific CDS tools may not be analytically feasible due to the relatively small size of the data sets that would be used for development. Therefore, there is a critical need to determine whether it is better to have multiple tools developed for specific patient populations, or more generalized tools that perform well—though perhaps not optimally—across multiple populations and environments.

Pediatric patients are an important subgroup for which it may be necessary to develop specialized CDS tools. It is well recognized that children have different physiological profiles, risk factors, and event rates for different clinical outcomes and adverse events [2–7]. Moreover, there are well documented differences in patterns of healthcare utilization and outcomes between adult and pediatric populations. Because many hospitals serve both pediatric and adult patients, it is necessary to determine whether CDS tools should be built specifically for different age groups versus for all patients. To date, the CDS tools that have been developed using adult data and then applied to children have not performed well [8–12]. Moreover, various pediatric-specific CDS tools or risk indices have been developed to reasonable levels of success, particularly when using modern predictive techniques incorporating machine learning [13–17].

At the beginning of the COVID-19 pandemic, we were tasked with developing a CDS tool for hospital resource utilization after planned elective surgeries, including anticipated length of stay, discharge to a skilled nursing facility, intensive care unit (ICU) admission, requirement for mechanical ventilation) [18]. Since the start of the pandemic surgical leadership has used the CDS tool in conjunction with knowledge of the local COVID-19 infection rates to determine whether to continue with or postpone an elective surgical case if the tertiary care hospital were to become resource-constrained. The data are pulled directly from our Epic-based system into a datamart. An R-script generates the requisite predictions, which are then visualized within a Tableau dashboard. In order to deploy the CDS quickly, we designed our tool to operate across all age groups and implemented it within a Tableau dashboard that has been in use since June 2020. Since implementing the CDS, we have had the opportunity to examine the applicability of the tool within the pediatric patient population. Herein, we directly compare the performance of two sets of CDS tools designed to predict post-surgical resource utilization: one trained on a mixed adult-pediatric data set, the other trained solely on pediatric data set. Additionally, we examine whether a machine-learning algorithm that is equipped to model heterogeneity (i.e., interactions of different characteristics) is better suited to operate across patient populations than a model that cannot model these interactions. Overall, our results show that while children have different risk factors than adults, machine-learning approaches are well suited to modeling these heterogeneities in a mixed sample.

## Methods

### Data

#### Study setting

This study was conducted using data from the electronic health records (EHR) system at Duke University Health System, which consists of three hospitals – a large tertiary care hospital and two community hospitals. Pediatric surgeries are almost exclusively performed at the tertiary care center. Our institution has used an integrated EPIC system since 2014, which covers the three hospitals in our system as well as a network of over 100 primary care and outpatient specialty clinics.

#### Cohort

We abstracted patient and encounter data for all elective surgeries from January 1, 2017, to March 1, 2020 (i.e., prior to the COVID-19 pandemic). There is no formal specification within our EHR for elective surgery. Instead, we included procedures coded with the admission source “Surgery Admit Inpatient.” This code corresponds to instances where the patient is admitted directly to the hospital for surgery rather than via, for example, via the emergency department. Additionally, we excluded procedures taking place on a Saturday or Sunday and any procedures that were not marked as completed. We defined a pediatric patient as any patient less than or equal to 18 years old on the date of their surgery. Patients were considered adults if they were 19 years of age or older at the time of surgery. We developed two cohorts for the purposes of model development. The “combined” cohort included all patients, regardless of age. The “pediatric” cohort excluded patients 19 years of age or older.

#### Predictor variables

We abstracted patient-level predictor variables known prior to the time of surgery, including patient demographics, service utilization history, medications prescribed in the past year, comorbidities, and surgery-specific factors. We abstracted pre-surgical CPT codes and grouped them by specialty. We retained all codes that had at least 25 total instances, resulting in 284 unique procedure groupings. A total of 53 unique predictor variables (with multiple levels each) were considered (Additional file 1: Tables 1 and 2). For binary variables such as comorbidities and medications, this list was winnowed such that each model used predictors present in at least 0.5% of cases. These binary predictors were calculated separately for the combined and pediatric cohorts. The model based on the combined cohort used 48 predictor variables, while the model based on the pediatric cohort used 34 predictor variables.

#### Outcome variable definition

In the initial development of the CDS tool, we were tasked with predicting four outcomes related to hospital resource utilization: overall length of stay, admission to the intensive care unit (ICU), requirement for mechanical ventilation, and discharge to a skilled nursing facility. Because children are rarely discharged to a skilled nursing facility and evaluating continuous outcomes poses unique challenges, we focused on the two binary outcomes: admission to the ICU and requirement for mechanical ventilation.

### Statistical Analysis

#### Descriptive statistics

We compared the pediatric and adult patient populations. We report standardized mean differences (SMDs) where an SMD > 0.10 indicates that the two groups are out of balance.

#### Predictive model algorithms

A predictive model may not transport well from one patient group to another if each group has different underlying risk factors for the outcomes of interest. Analytically, this would mean that there is an interaction between a demographic characteristic (i.e., age) and a risk factor (e.g., weight). To assess this hypothesis, we considered three modeling approaches. In our initial work we used the Random Forests (RF) algorithm [19]. RF is a machine-learning algorithm that consists of an aggregation of decision trees; one feature of decision trees is that they are well suited for modeling interactions. The second approach was LASSO logistic regression. LASSO is an extension of logistic regression that performs an implicit variable selection to generate more stable predictions [20]. Like typical regression models, LASSO does not explicitly model interactions. Our final model was also a LASSO model to which we explicitly added an interaction term between age and each predictor.

#### Analysis workflow

**Fig. 1:**
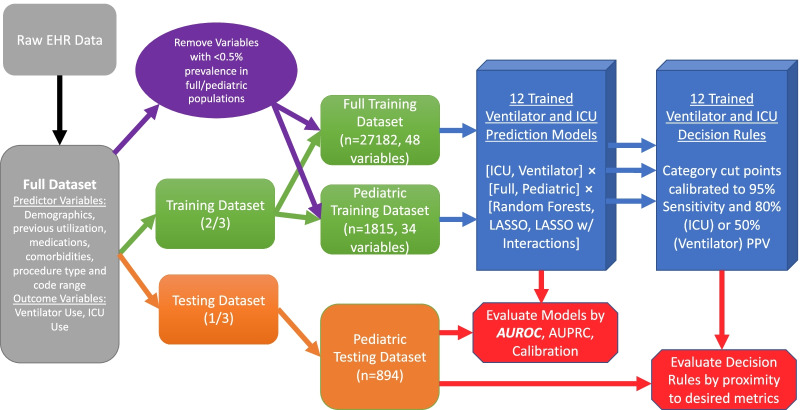
Workflow for training and validating the predictive moddels

Our overall workflow is shown in Fig. 1. We randomly divided the full dataset into training (two-thirds) and testing (one-third) sets. From the training set, we created two analytic training cohorts: a combined dataset of adults and children and a subset of children alone. For the testing set, we only used children to assess how the different models perform in a pediatric population. We fit the models on the training data using cross-validation to choose optimal tuning parameters and applied the best model to the independent test data. Overall, we fit a total of 12 models that combined two outcomes, two cohorts (combined and pediatric), and three modeling approaches. To assess performance during the COVID period, we abstracted data on pediatric encounters March 2020 to January 2022.

#### Model metrics

For each of the 12 models we calculated global performance metrics, including the area under the receiver operator characteristic (AUROC) and the calibration slope. We used a bootstrap to calculate 95% confidence intervals and a permutation test to assess differences between the model AUROCs. To gain insights into the differences between the combined and pediatric models, we used the RF model fit to identify the top important variables within each model.

#### Decision rule analysis

We assessed the impact each model would have on a generated decision rule. As in our initial CDS tool, we transformed the predicted probabilities into discrete categorizations of low, medium and high risk. The lower cutoff was calibrated in all models to correspond to a sensitivity of 95%, such that 95% of the positive training data fell into the medium- or high-risk categories and 5% into the low-risk category. This focus on sensitivity was intended to overestimate rather than underestimate risk in assigning categories. The upper cutoff was set to maximize the utility of the high-risk category, thereby creating a model with a large positive predictive value (PPV = true positives/predicted positives) while still encompassing a significant portion of the data. Because of the large difference in baseline probability between ICU admission and requirement for mechanical ventilation, this threshold was set separately for each outcome. We determined that an 80% PPV was optimal for models predicting ICU admission and a 50% PPV was optimal for models predicting requirement for mechanical ventilation.

All analyses were performed in R version 3.6.3. The ranger and glmnet packages were used for the RF and LASSO models, respectively [21, 22]. This work was declared exempt by the DUHS IRB.

## Results

### Cohort description

We abstracted data on a total of 42,209 elective surgeries, of which 39,547 (94%) were for patients 19 years of age and older and 2,662 (6%) patients 18 years of age or younger. Table 1 presents patient information stratified on age. As expected, there were meaningful differences (SMD > 0.10) for almost all patient characteristics, highlighting the differences between adult and pediatric patients. Table 2 shows surgery characteristics stratified on age, similarly showing meaningful differences in resource utilization, severity, and procedure type.

**Table 1:** Characteristics of the patient cohort

Patient characteristics	Pediatric patients (< = 18 years of age) N = 2792	Adult patients (> = 19 years of age) N = 39,417	Standardized difference
Age (Mean, SD)	8.26 (6.25)	61.0 (14.1)	4.822*
Sex (N, %)			
Male	1433 (51.3%)	18,452 (46.8%)	0.090
Female	1359 (48.7%)	20,965 (53.2%)	
Race (N, %)			
Non-Hispanic white	1555 (55.7%)	29,219 (74.1%)	0.501*
Non-Hispanic black	615 (22.0%)	7662 (19.4%)	
Hispanic	265 (9.5%)	728 (1.8%)	
Other	357 (12.8%)	1808 (4.6%)	
Height (Mean, SD)	48.1 (15.8)	66.9 (4.15)	1.626*
Weight (Mean, SD)	1260 (1010)	3110 (809)	2.021*
BMI (N, %)			
Underweight	1417 (50.8%)	366 (0.9%)	1.973*
Normal	981 (35.1%)	8377 (21.3%)	
Overweight	176 (6.3%)	12,639 (32.1%)	
Obese	188 (6.7%)	18,013 (45.7%)	
None	30 (1.1%)	22 (0.1%)	
Previous healthcare utilization (Mean, SD)			
Hospital encounters	0.602 (1.49)	0.276 (0.749)	0.277*
Ambulatory encounters	13.9 (15.8)	17.2 (18.8)	0.192*
Emergency department encounters	0.219 (1.92)	0.204 (0.970)	0.009
Comorbidities (Top 15: N, %)			
Cardiovascular disease	870 (31.2%)	23,713 (60.2%)	0.609*
Psychiatric disease	614 (22.0%)	19,117 (48.5%)	0.578*
Hypertension	152 (5.4%)	17,722 (45.0%)	1.022*
Diabetes	20 (0.7%)	2973 (17.7%)	0.614*
Atherosclerotic CVD	2 (0.1%)	5133 (13.0%)	0.542*
Coronary artery disease	1 (0.0%)	4589 (11.6%)	0.511*
COPD	13 (0.5%)	2764 (7.0%)	0.350*
AFIB	0 (0.0%)	2405 (6.1%)	0.360*
Congestive heart failure	16 (0.6%)	1551 (3.9%)	0.228*
Peripheral vascular disease	12 (0.4%)	1511 (3.8%)	0.237*
Diabetic renal	3 (0.1%)	1375 (3.5%)	0.257*
CVA/TIA	56 (2.0%)	963 (2.4%)	0.030
Liver disease	9 (0.3%)	938 (2.4%)	0.179*
Pulmonary hypertension	54 (1.9%)	692 (1.8%)	0.013
End-stage renal disease	13 (0.5%)	496 (1.3%)	0.086
Concurrent medications (Top 15: N, %)			
Statins	3 (0.1%)	14,408 (36.6%)	1.068*
Antiplatelet	139 (5.0%)	13,383 (34.0%)	0.786*
Opioid	128 (4.6%)	11,768 (29.9%)	0.710*
Diuretics	175 (6.3%)	11,116 (28.2%)	0.607*
Hypertension medication	126 (4.5%)	10,394 (26.4%)	0.635*
Beta blocker	46 (1.6%)	9757 (24.8%)	0.726*
Anti-arrhythmic	39 (1.4%)	8577 (21.8%)	0.671*
ACE inhibitor	65 (2.3%)	7935 (20.1%)	0.588*
Calcium channel blocker	47 (1.7%)	7822 (19.8%)	0.613*
Angiotensin receptor blocker	14 (0.5%)	6320 (16.0%)	0.588*
Anticoagulant	32 (1.1%)	2976 (7.6%)	0.318*
Insulin	8 (0.3%)	2859 (7.3%)	0.372*
Oral diabetic	0 (0.0%)	2592 (6.6%)	0.375*
Nitrates	0 (0.0%)	2031 (5.2%)	0.330*
Digoxin	31 (1.1%)	122 (0.3%)	0.095

^*^Standardized difference greater than 0.10 indicates meaningful difference between cohorts

**Table 2:** Elective surgery characteristics and resource utilization

Surgery characteristics	Pediatric patients (< = 18 years of age) N = 2792	Adult patients (> = 19 years of age) N = 39,417	Standardized difference
Post-surgery resources			
Hospital length of stay (Mean, SD)	5.58 (13.8)	3.44 (4.63)	0.208 *
ICU admission (N, %)	1020 (36.5%)	5402 (13.7%)	0.546*
Required mechanical ventilation (N, %)	241 (8.6%)	1388 (3.5%)	0.215*
Procedure severity			
Minor	181 (6.5%)	1515 (3.8%)	0.754*
Moderate	233 (8.3%)	8251 (20.9%)	
Major	1012 (36.2%)	22,500 (57.1%)	
None	1366 (48.9%)	7151 (18.1%)	
Top 10 Adult surgical procedures (by Primary CPT Code)			
Total knee arthroplasty (27,447)	0 (0.0%)	3539 (9.0%)	0.932*
Total hip arthroplasty (27,130)	9 (0.3%)	3254 (8.3%)	
Total shoulder arthroplasty (23,472)	1 (0.0%)	1226 (3.1%)	
Anterior arthrodesis incl. cervical discectomy below C2 (225,510)	2 (0.1%)	1160 (2.9%)	
Microsurgical w/ microscope (69,990)	191 (6.8%)	921 (2.3%)	
Lumbar arthrodesis w/ posterior technique (22,633)	5 (0.2%)	871 (2.2%)	
Anterior interbody arthrodesis incl. minimal discectomy (22,558)	2 (0.1%)	780 (2.0%)	
Autologous Blood Collection (86,891)	28 (1.0%)	747 (1.9%)	
Laparoscopy w/ gastric bypass and roux-en-Y (43,644)	4 (0.1%)	736 (1.9%)	
Intervertebral insertion of biomechanical device (22,853)	0 (0.0%)	651 (1.7%)	
Other	2550 (91.3%)	25,532 (64.8%)	
Top 10 pediatric surgical procedures (by primary CPT code)			
Microsurgical w/ microscope (69,990)	191 (6.8%)	921 (2.3%)	0.836*
Posterior arthrodesis for spinal deformity, 7–12 segments (22,802)	190 (6.8%0	114 (0.3%)	
Posterior spinal instrumentation, > 13 segments (22,844)	118 (4.2%)	121 (0.3%)	
Fluoroscopy for placement of central venous access device (77,001)	108 (3.9%)	8 (0.0%)	
Laparoscopic gastrostomy without construction of gastric tube (43,653)	60 (2.1%)	7 (0.0%)	
Remove and replace cerebrospinal fluid shunt (62,258)	58 (2.1%)	24 (0.1%)	
Negative pressure wound therapy (97,605)	50 (1.8%)	179 (0.5%)	
Subtrochanteric osteotomy with internal fixation (27,165)	43 (1.5%)	3 (0.0%)	
Other craniofacial/maxillofacial (21,299)	42 (1.5%)	0 (0.0%)	
Reimplant single ureter (50,780)	42 (1.5%)	5 (0.0%)	
Other	1890 (67.7%)	38,035 (96.5%)	

^*^Standardized difference greater than 0.10 indicates meaningful difference between cohorts

### Predictive model performance

We built 6 models for each of the two clinical outcomes of interest – ICU admission and requirement for mechanical ventilation. After building models based on their respective training sets (n = 27,182 for combined adult and pediatric patients; n = 1,815 for pediatric patients only), we evaluated all models against the pediatric testing set (n = 894; results shown in Table 3). The best prediction model for ICU admission had an AUROC of 0.945 (95% CI: 0.928, 0.960) in the test data while the best model for requirement for mechanical ventilation had an AUROC of 0.862 (95% CI: 0.919, 0.902). ROC plots are shown in Additional file 1: Fig. 1a and b. There were significant differences in performance between the different models when using the combined adult/pediatric data. In predicting ICU admission and requirement for mechanical ventilation, the RF models performed better overall than the LASSO models (ICU admission: *p* < 0.001; requirement for mechanical ventilation: *p* < 0.023). Performance was not significantly different between the RF models using combined adult/pediatric data and pediatric data alone (ICU admission: *p* = 0.886; requirement for mechanical ventilation: *p* = 0.112). Conversely, the performance of the pediatric LASSO models was significantly better than LASSO models developed with combined adult/pediatric data (ICU admission: *p* < 0.002; requirement for mechanical ventilation: *p* < 0.028). Incorporation of explicit age-based interactions in the LASSO model attenuated differences between models developed with combined adult/pediatric data and pediatric data alone, and there was only a significant difference in performance for models predicting ICU admission (ICU admission *p* < 0.004; requirement for mechanical ventilation *p* = 0.077). Testing the model on 1428 pediatric encounters during the COVID period yielded very similar performance (Additional file 1: Table 3).

**Table 3:** Model performance for each algorithm and outcome

	ICU		Ventilator	
	Combined	Pediatric	Combined	Pediatric
Random forests				
AUROC	0.942 [0.925, 0.958]	0.945 [0.928, 0.960]	0.862 [0.819, 0.902]	0.851 [0.807, 0.893]
Calibration	1.414 [1.203, 1.701]	1.374 [1.161, 1.642]	0.935 [0.778, 1.131]	0.894 [0.743, 1.090]
Low sensitivity	0.988	0.967	0.988	0.953
High PPV	0.865	0.856	0.355	0.461
LASSO				
AUROC	0.911 [0.890, 0.930]	0.930 [0.911, 0.949]	0.821 [0.765, 0.872]	0.860 [0.820, 0.898]
Calibration	0.926 [0.807, 1.082]	1.071 [0.946, 1.245]	0.673 [0.527, 0.848]	0.826 [0.680, 0.999]
Low sensitivity	0.979	0.935	0.976	0.941
High PPV	0.838	0.786	0.335	0.434
LASSO interactions				
AUROC	0.917 [0.897, 0.936]	0.932 [0.911, 0.950]	0.838 [0.795, 0.879]	0.860 [0.817, 0.898]
Calibration	0.980 [0.853, 1.146]	0.993 [0.871, 1.166]	0.813 [0.671, 0.9876]	0.952 [0.789, 1.138]
Low sensitivity	0.976	0.885	0.976	0.918
High PPV	0.845	0.769	0.346	0.418

ICU: Intensive care unit; AUROC: area under the receiver operator characteristic; PPV: positive predictive value

[Bracketed values represent 95% confidence intervals]

### Decision rule performance

**Fig. 2:**
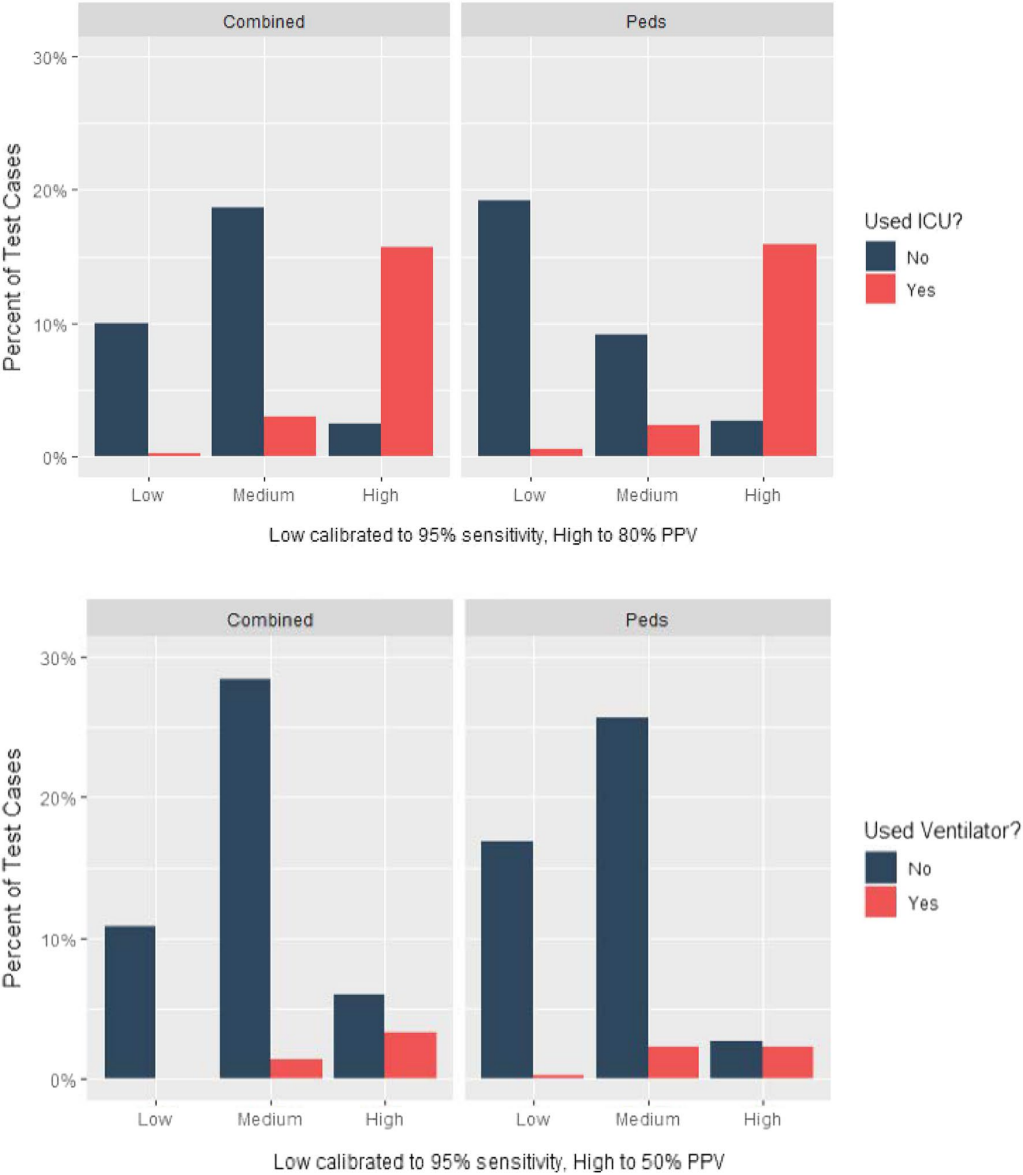
Performance of random forests cutpoints on test data. Performance of a decision rule for each outcome and cohort. The pediatric model is better able to obtain the nominal target of 95% sensitivity for medium/high risk patients along with the desired positive predictive value

We assessed the accuracy of decision rules for each model using the pediatric test data (Fig. 2). We set the desired sensitivity of the low threshold at 95%. We set the PPV of ICU admission and requirement for mechanical ventilation at 80% and 50%, respectively. While the RF models showed no difference in global performance (based on AUROC), there were slight differences in the performance of a decision rule (Table 3). Importantly, the models developed using only pediatric data were closer to the desired decision rule metrics than models developed using combined adult/pediatric data for prediction of both ICU admission and requirement for mechanical ventilation. Of note, the models trained on combined adult/pediatric data were more sensitive than the models trained on pediatric data alone for both ICU admission (0.979 vs. 0.935) and requirement for mechanical ventilation (0.976 vs. 0.941).

### Variable importance

While RF does not generate beta coefficients, this machine learning approach can identify “important” predictors, or predictors that play a role in achieving the prediction accuracy of the model. Table 4 shows the top 10 variables identified by the RF models for both combined adult/pediatric and purely pediatric cohorts. All four models included age, height, weight, previous ambulatory encounters, specialty, and service among the top predictors. Notably, the important predictors for the different models varied with respect to the specific surgery CPT code, comorbidities, and medication usage.

**Table 4:** Top important variables from each model

variable rank	Ventilator		ICU	
	Combined RF	Pediatric RF	Combined RF	Pediatric RF
1	Specialty	Specialty	CPT 33,361–33,496 Surgical Procedures on Aortic Valve	Specialty
2	Service	Service	CPT 33,510–33,536 Venous Grafting for Coronary Artery Bypass	Weight
3	CPT 69,990–69,990 Operating Microscope Procedures	Height	CPT 33,508–33,508 Endoscopy Surrounding Vein for Coronary Artery Bypass	Height
4	CPT 61,510–61,516 Craniectomy or Craniotomy Procedures	Weight	Marker for Cardiac Surgery	Service
5	CPT 33,361–33,496 Surgical Procedures on Aortic Valve	History of Cardiovascular Disease	Specialty	Age
6	Weight	CPT 33,608–33,681 Repair Procedures for Single Ventricle or Cardiac Anomalies	Weight	Previous ambulatory encounters
7	Age	Age	Service	Diuretics
8	Height	Previous ambulatory encounters	Height	CPT 33,608–33,681 Repair Procedures for Single Ventricle or Cardiac Anomalies
9	Previous ambulatory encounters	CPT 61,343–61,343 Craniectomy for Decompression	Age	History of Cardiovascular Disease
10	CPT 20,650–20,664 Introduction or Removal Procedures on Musculoskeletal System	CPT 61,760–61,793 Stereotaxis Procedures on Skull, Meninges, and Brain	Previous ambulatory encounters	Previous Hospital Encounters

RF: Random forests

## Discussion and conclusion

We sought to assess the performance of CDS tools to predict resource utilization after pediatric elective surgeries using either combined adult/pediatric data or pediatric data alone. Our results indicate that models using a traditional regression-based method exhibit better performance if they are built with cohort specific pediatric data than with combined adult/pediatric data. In contrast, models using a machine learning method (RF) exhibited better performance when built using combined adult/pediatric data than with pediatric data alone. These findings suggest that machine learning-based models may be able to more appropriately account for key differences between pediatric and adult patient populations. Moreover, these findings have important implications for the development of CDS tools for different populations.

CDS tools are increasingly used to help guide clinical care and decision making; however, very few of these tools are developed specifically for pediatric populations [23]. In our experience, data from pediatric patients are frequently removed from the datasets used to develop CDS tools. Moreover, datasets for specific clinical subpopulations, such as pediatric patients, are frequently not large enough to train the models that underlie these tools. Further, it is valuable to be able to use a single CDS across multiple patient populations. For example, while tools exist for hospital readmission for specific sub-populations [24, 25], we have found it easier to use a generalized hospital readmission risk score at our own institution [26]. Being able to leverage data from both adult and pediatric patients in the development of CDS tools could facilitate the development of CDS tools that perform well among pediatric patient populations.

Though we found that CDS tools based on combined adult and pediatric data perform well, this does not imply that children and adults have the same risk factors. Other groups have found that models trained solely on adults do not translate well to children [8], including tools for comorbidity indices [9], emergency medical services (EMS) dispatch triage protocols [10], mortality scores [11], and surgery duration [12]. Similarly, we found that models built with regression-based methods using combined adult and pediatric data did not perform as well within the pediatric population as a model built on pediatric data alone. Importantly, a machine learning method that included interaction terms for age improved the transportability of a regression-based model developed with combined adult and pediatric data to a pediatric population. These findings are supported by the examination of the top predictor variables from the models using combined adult and pediatric data versus pediatric data alone, in which adult and pediatric populations were found to have different important predictors for the clinical outcomes of interest. Our findings demonstrate that accurately capturing and modeling differences associated with clinical sub-populations is a critical component in developing transportable CDS tools.

Our study has several strengths as well as some limitations. We leveraged a large dataset from a diverse patient population to develop and test multiple modeling approaches. However, as a single center study, model development and performance are dependent on local context, including composition of the local patient population, types of data commonly captured within the EHR, and the clinical scenario for which the CDS tool is being developed. Moreover, study results are likely to be dependent on the relative size of the pediatric population available for model development. It is possible that a health system with a high volume of pediatric surgeries may have a pediatric population that is large enough to develop a CDS tool specific to that population. Our findings are also specific to CDS tools designed to predict post-surgical resource utilization and may or may not be generalizable to other CDS types. Therefore, we do not view these as definitive results that model developers should always combine adult and pediatric data. Instead, our study provides an overall approach that can be used to develop and evaluate different models for CDS tools.

Overall, our findings demonstrate that while adults and children have different risk factors and characteristics that are important for predicting clinical outcomes, appropriate machine learning techniques can generate CDS tools that effectively model outcomes for both pediatric and adult populations. Importantly, this finding suggests that even for clinical outcomes for which the relevant pediatric patient population may be small, models may be developed using data from adults, provided that the model accounts for the interaction between age and important patient- and procedure-level factors. Further, these findings indicate that there are additional opportunities and clinical scenarios that may be amenable to the development and application of CDS tools.

## Supplementary information

**Additional file 1: Supplemental Table 1.** Predictor Variables used in each model. **Supplemental Table 2.** CPT Code Ranges Used (286, including None and Other). **Supplemental Table 3.** Performance of models on pediatric patients (n = 1428) during the COVID period (March 2020 – January 2022). **Supplemental Figure 1.** ROC plots for ICU and Ventilator models based on training population and model type.

## Data Availability

The datasets generated and/or analyzed during the current study are not publicly available due patient privacy but are available from the corresponding author on reasonable request.
