# Correction to: Combining adult with pediatric patient data to develop a clinical decision support tool intended for children: leveraging machine learning to model heterogeneity

**DOI:** 10.1186/s12911-022-01846-1

**Published:** 2022-05-12

**Authors:** Paul Sabharwal, Jillian H. Hurst, Rohit Tejwani, Kevin T. Hobbs, Jonathan C. Routh, Benjamin A. Goldstein

**Affiliations:** 1 Department of Computer Science, Duke University, Durham, NC USA; 2 Children’s Health and Discovery Initiative, Department of Pediatrics, Duke University, Durham, NC USA; 3 Division of Infectious Diseases, Department of Pediatrics, Duke University, Durham, NC USA; 4 Division of Urology, Department of Surgery, Duke University, Durham, NC USA; 5 Department of Biostatistics and Bioinformatics, Duke University, 2424 Erwin Road, Durham, NC 27705 USA

## Correction to: BMC Medical Informatics and Decision Making (2022) 22:84 10.1186/s12911-022-01827-4

Following publication of the original article [1], it was reported that part of the ‘Outcome Variable Definition’ and the entirety of the ‘Descriptive statistics’ subsection was missing. These two subsections are given below with the previously missing text highlighted in bold. The original article [1] has been updated.

Outcome Variable Definition

In the initial development of the CDS tool, we were tasked with predicting four outcomes related to hospital resource utilization: overall length of stay, admission to the intensive care unit (ICU), requirement for mechanical ventilation, and discharge to a skilled nursing facility. **Because children are rarely discharged to a skilled nursing facility and evaluating continuous outcomes poses unique challenges, we focused on the two binary outcomes: admission to the ICU and requirement for mechanical ventilation.**

**Statistical Analysis**

***Descriptive statistics***

**We compared the pediatric and adult patient populations. We report standardized mean differences (SMDs) where an SMD > 0.10 indicates that the two groups are out of balance.**
