# Supplementary material for: Combining adult with pediatric patient data to develop a clinical decision support tool intended for children: leveraging machine learning to model heterogeneity
Source: BMC Med Inform Decis Mak. 2022 Mar 29;22:84. doi: 10.1186/s12911-022-01827-4 (PMC8961261; doi:10.1186/s12911-022-01827-4)
Supplement: Supplementary file 1 — Additional file 1: Supplemental Table 1. Predictor Variables used in each model. Supplemental Table 2. CPT Code Ranges Used (286, including None and Other). Supplemental Table 3. Performance of models on pediatric patients (n = 1428) during the COVID period (March 2020 – January 2022). Supplemental Figure 1. ROC plots for ICU and Ventilator models based on training population and model type. [file 12911_2022_1827_MOESM1_ESM.docx]

Supplemental Material:

**Supplemental Table 1: Predictor Variables used in each model**

| **Used in Both Models (33)** | **Only in Full Model (15)** | **Only Pediatric Model (1)** | **Neither Model (4)** |
| --- | --- | --- | --- |
| Age | Smoker | Digoxin | Heart Failure |
| Sex | COPD |  | Pulmonary Hypertension Medication |
| Race | Myocardial Infarction |  | Prostacyclin |
| BMI | Peripheral Vascular Disease |  | Endothelin Receptor Agonists |
| Height | AFIB |  |  |
| Weight | Atherosclerotic CVD |  |  |
| Previous Hospital Encounters | Coronary Artery Disease |  |  |
| Previous Ambulatory Encounters | Diabetic Renal |  |  |
| Previous Emergency Department Encounters | End-State Renal Disease |  |  |
| Surgery Specialty | Nitrates |  |  |
| Service of Surgery | Statins |  |  |
| Day of Week | Oral Diabetic Medication |  |  |
| Procedure Severity Code | Insulin |  |  |
| CPT Code Range | Liver Disease |  |  |
| Psychiatric Disease | PDE 5 Inhibitors |  |  |
| Diabetes |  |  |  |
| Congestive Heart Failure |  |  |  |
| Hypertension |  |  |  |
| CVA/TIA |  |  |  |
| Cardiovascular Disease |  |  |  |
| Pulmonary Hypertension |  |  |  |
| Stent |  |  |  |
| Cardiac Surgery |  |  |  |
| Hypertension Medication |  |  |  |
| ACE Inhibitors |  |  |  |
| Angiotensin Receptor Blocker |  |  |  |
| Beta Blocker |  |  |  |
| Calcium Channel Blocker |  |  |  |
| Diuretics |  |  |  |
| Opioid |  |  |  |
| Anticoagulant |  |  |  |
| Antiplatelet |  |  |  |
| Antiarrhythmic |  |  |  |

**Supplemental Table 2: CPT Code Ranges Used (286, including None and Other)**

| 10180-10180 | 11000-11047 | 11100-11107 | 11200-11646 | 11960-11971 | 11977-11981 | 11982-11982 |
| --- | --- | --- | --- | --- | --- | --- |
| 12001-13133 | 14000-15738 | 15755-15776 | 15777-15787 | 15860-15879 | 19260-19272 | 19303-19307 |
| 19316-19380 | 20200-20251 | 20650-20664 | 20665-20697 | 20900-20902 | 20926-20926 | 20930-20931 |
| 20936-20939 | 20955-20973 | 21013-21016 | 21025-21050 | 21120-21208 | 21299-21299 | 21300-21495 |
| 21600-21633 | 21705-21705 | 21800-21825 | 22010-22015 | 22206-22230 | 22510-22515 | 22532-22812 |
| 22830-22830 | 22840-22855 | 23430-23440 | 23470-23474 | 23500-23680 | 26990-26991 | 27000-27001 |
| 27005-27027 | 27030-27033 | 27065-27079 | 27090-27091 | 27125-27138 | 27146-27147 | 27156-27158 |
| 27161-27170 | 27175-27187 | 27230-27269 | 27299-27299 | 27301-27301 | 27350-27360 | 27364-27364 |
| 27365-27365 | 27372-27400 | 27437-27447 | 27448-27468 | 27486-27487 | 27488-27488 | 27495-27495 |
| 27500-27519 | 27520-27566 | 27570-27580 | 27590-27598 | 27600-27606 | 27635-27647 | 27650-27692 |
| 27695-27698 | 27700-27703 | 27705-27715 | 27720-27726 | 27750-27848 | 27870-27871 | 27880-27889 |
| 28300-28312 | 28705-28735 | 28740-28750 | 28800-28825 | 29000-29590 | 29914-29916 | 29999-29999 |
| 31251-31259 | 31271-31299 | 31360-31382 | 31515-31526 | 31535-31536 | 31600-31610 | 31622-31629 |
| 31636-31646 | 32200-32320 | 32440-32501 | 32550-32551 | 32601-32602 | 32605-32606 | 32607-32609 |
| 32650-32656 | 32662-32662 | 32663-32663 | 32666-32672 | 32674-32674 | 33015-33130 | 33200-33249 |
| 33250-33261 | 33361-33496 | 33508-33508 | 33510-33536 | 33608-33681 | 33692-33730 | 33750-33768 |
| 33820-33853 | 33860-33886 | 33910-33917 | 34490-34510 | 34705-34705 | 34706-34826 | 34841-34900 |
| 35111-35162 | 35301-35301 | 35355-35381 | 35537-35558 | 35565-35571 | 35646-35661 | 35665-35671 |
| 36493-36510 | 36555-36573 | 36800-36821 | 36825-36830 | 37215-37216 | 37217-37249 | 38100-38129 |
| 38220-38222 | 38300-38780 | 38790-38792 | 38794-38999 | 39000-39220 | 39400-39402 | 39545-39599 |
| 41116-41155 | 41874-41899 | 42104-42340 | 42408-42510 | 42820-42836 | 43040-43136 | 43197-43212 |
| 43234-43242 | 43279-43280 | 43281-43282 | 43283-43288 | 43289-43289 | 43332-43337 | 43620-43634 |
| 43644-43645 | 43653-43653 | 43659-43659 | 43770-43775 | 43800-43825 | 43999-43999 | 44005-44005 |
| 44015-44015 | 44120-44128 | 44130-44130 | 44139-44160 | 44180-44180 | 44186-44187 | 44188-44188 |
| 44202-44203 | 44204-44208 | 44210-44213 | 44227-44227 | 44238-44239 | 44300-44316 | 44320-44345 |
| 44620-44660 | 44950-44979 | 45110-45136 | 45300-45305 | 45308-45331 | 45395-45397 | 45990-45990 |
| 47120-47134 | 47300-47399 | 47562-47564 | 47600-47630 | 47701-47801 | 48105-48148 | 45150-48150 |
| 45152-48154 | 48556-48999 | 49000-49010 | 49185-49215 | 49320-49322 | 49324-49329 | 49412-49423 |
| 49491-49535 | 49560-49611 | 49652-49659 | 50010-50081 | 50220-50240 | 50543-50543 | 50544-50544 |
| 50545-50548 | 50605-50605 | 50610-50660 | 50715-50945 | 51005-51080 | 51101-51597 | 51720-51720 |
| 52000-52000 | 52005-52005 | 52214-52240 | 52332-52332 | 52351-52351 | 52353-52353 | 52601-52601 |
| 53210-53431 | 55801-55845 | 55866-55866 | 56520-56700 | 57106-57112 | 57230-57270 | 57287-57288 |
| 57410-57410 | 55140-58146 | 58150-58294 | 58570-58575 | 58660-58660 | 58661-58661 | 58720-58720 |
| 58953-58956 | 60210-60271 | 60280-60545 | 60650-60699 | 61333-61055 | 61250-61315 | 61343-61343 |
| 61450-61460 | 61503-61501 | 61510-61516 | 61518-61530 | 61531-61533 | 61532-61545 | 61546-61548 |
| 61550-61552 | 61582-61596 | 61597-61597 | 61598-61608 | 61615-61616 | 61680-61711 | 61750-61751 |
| 61760-61793 | 61796-61875 | 62100-62148 | 62160-62160 | 62161-62164 | 62165-62165 | 62180-62258 |
| 62270-62272 | 62310-62350 | 62380-63252 | 63265-63308 | 64486-64550 | 64553-64647 | 64702-64727 |
| 64732-64792 | 64802-64999 | 69433-69440 | 69990-69990 | 72010-72120 | 74415-74710 | 75705-75746 |
| 75998-76066 | 76981-76999 | 77001-77003 | 77011-77014 | 86828-86911 | 93303-93355 | 95857-95872 |
| 95885-95943 | 95950-95962 | 95965-95984 | 97597-97608 | None | Other |  |

**Supplemental Table 3: Performance of models on pediatric patients (n = 1428) during the COVID period (March 2020 – January 2022)**

|  | | **ICU** | | **Ventilator** | |
| --- | --- | --- | --- | --- | --- |
|  |  | **Combined** | **Peds** | **Combined** | **Peds** |
| **Random Forests** | **AUROC** | 0.936  [0.923, 0.949] | 0.936  [0.921, 0.948] | 0.902  [0.871, 0.929] | 0.887  [0.856, 0.915] |
|  | **Calibration** | 1.236  [1.098, 1.409] | 1.251  [1.114, 1.405] | 1.183  [1.026, 1.374] | 1.124  [0.972, 1.302] |
|  | **Low Sensitivity** | 0.992 | 0.946 | 1.000 | 0.982 |
|  | **High PPV** | 0.822 | 0.813 | 0.371 | 0.514 |
| **LASSO** | **AUROC** | 0.902  [0.885, 0.918] | 0.922  [0.906, 0.936] | 0.860  [0.827, 0.895] | 0.864  [0.825, 0.901] |
|  | **Calibration** | 0.863  [0.780, 0.960] | 0.983  [0.898, 1.089] | 0.915  [0.789, 1.057] | 0.989  [0.851, 1.169] |
|  | **Low Sensitivity** | 0.971 | 0.919 | 0.991 | 0.954 |
|  | **High PPV** | 0.772 | 0.726 | 0.328 | 0.430 |
| **LASSO Interactions** | **AUROC** | 0.909  [0.893, 0.923] | 0.926  [0.911, 0.940] | 0.863  [0.824, 0.898] | 0.865  [0.824, 0.905] |
|  | **Calibration** | 0.908  [0.825, 1.004] | 1.008  [0.925, 1.104] | 1.044  [0.892, 1.216] | 1.103  [0.939, 1.292] |
|  | **Low Sensitivity** | 0.986 | 0.890 | 0.982 | 0.917 |
|  | **High PPV** | 0.764 | 0.741 | 0.339 | 0.436 |
| ICU: Intensive Care Unit  AUROC: Area Under the Receiver Operator Characteristic  PPV: Positive Predictive Value  [Bracketed values represent 95% confidence intervals] | | | | | |
